# Risk factors associated with intimate partner violence (IPV) against Jordanian married women: A social ecological perspective

**DOI:** 10.1016/j.heliyon.2024.e30364

**Published:** 2024-04-26

**Authors:** Hussein Mohammad Alothman, Abdel Rahman Ahmed AbdelRahman, Semiyu Adejare Aderibigbe, Muhamed Ali

**Affiliations:** a Department of Sociology, College of Arts, Humanities, and Social Sciences, University of Sharjah, Sharjah, United Arab Emirates; b Department of International Relations, College of Arts, Humanities, and Social Sciences, University of Sharjah, Sharjah, United Arab Emirates; c College of Arts, Humanities and Social Sciences and Institute of Leadership in Higher Education, University of Sharjah, Sharjah, United Arab Emirates

**Keywords:** Intimate partner violence, Risk factors, Jordan

## Abstract

This paper seeks to gain insights into complex and multiple influences which may behind the different components of intimate partner violence (IPV) against Jordanian wives. Drawing on a quantitative approach, the paper explores and presents findings of the determinants of domestic violence sustained by female partners during the year preceding a nationally representative survey. The survey is based on national multi-stage random sampling data from the 2012 Jordan Demographic Health Survey (JDHS). The paper applies the social-ecological framework. A method of Leastwise deletion techniques is used to remove missing data. Doing this resulted in 6213 married women used in the data analysis. Logistic regression models are used to estimate/predict different forms of IPV against domestic female partners. The findings of the study suggest that wives are victimized at all levels of the framework. Specifically, family, community, and social levels were the most vital factors affecting victims experiencing IPV. More specifically, wives witnessing their fathers beat their mothers and wives who are scared of their husbands are more prone to sustain violence inflicted by their partners. Furthermore, wives' education, working status, and age at marriage do not predict IPV. Another key finding is that wife empowerment in family relationships is a protective factor against domestic violence against her. The implication is that the patriarchal explanation of domestic violence against wives is valid in the Jordanian cultural context. This study underscores the need to reevaluate the effectiveness of Jordan's general human development programs and women empowerment programs as an essential measure for alleviating IPV inflicted on married women in Jordan.

## 1 Introduction

IPV is a worldwide social phenomenon that is defined as “an intentional control or victimization of a person with whom the abuser has had or is currently in an intimate, romantic, or spousal relationship" [1]. Manifested in physical, emotional, and sexual forms, IPV against women severely affects their overall health and social well-being [2]. The Middle East is not an exception, as research indicates that the percentages of wives who have sustained physical IPV during their lifetime ranges from 8.1 % in Israel to 34.4 % in Egypt [3]. In 2007, physical, emotional, and sexual IPV affected approximately 12.2 %, 14 %, and 5.6 %, respectively, of Jordanian female partners aged 15–49 within 12 months preceding the survey [4]. Interestingly, in 2012, these percentages of physical and sexual IPV prevalence remained the same, whereas emotional IPV increased by about 3.4 % [5].

Globally, it is noted that relatively little research has been conducted on hazards and challenges resulting in IPV by integrating different frameworks [6]. For instance, one study in the West Bank and Gaza, using a national sample of wives, reported some causes of the 12-month incidence of violence orchestrated by male-to-female spouses [7]. Informed by representative national data, two previous research in Jordanian society examined women's experience of abuse [8]. While one examined demographic and violence history, the other explored demographic and economic opportunity causative conditions for IPV experience [9]. A few researchers conducted their studies based on a representative subgroup, such as the population living in Palestinian camps in Jordan [10] and the southern Jordan region [11]. Other researchers in Jordan dealt with IPV against women with non-representative data in clinical settings [12,13]. Thus, there is a need for more comprehensive and representative studies on the causes of domestic violence against Jordanian wives.

Our study aims to complement existing literature on IPV by focusing on the Jordanian context and exploring additional risk factors identified in the JDHS 2012. The study incorporates factors from the social-ecological model's different analytical levels. The model conceives domestic violence against women to be a complex question related to the various dimensions of human development [14]. Therefore, examining the problem through multiple perspectives can reveal valuable insights into IPV experiences, especially for women in Jordan. Section 2 below reviews some pertinent literature on the subject. Section 3 encompasses the methodology of the study. The last three sections of the paper focus on the study's findings, discussion, and conclusions.

## 2 Related literature

This section provides valuable insights into previous findings and their implications for understanding the issue being explored. Essentially, the section outlines the components of the social-ecological framework which is a broad conceptualization focusing on the different levels of macro and micro factors behind violence against wives. This model helps educators and researchers understand the complexities of real-life situations regarding the issue [14]. As discussed, violence against women is a multifaceted reality that involves different levels of human development, ranging from personal history and resources to cultural values and beliefs [14,15]. This model identifies four levels of analysis. The individual level examines the personal history, resources, and individual development experience that partners bring to their relationships [15]. The family level explores the role structure and interactional dynamics within the family [14]. The community level focuses on the community's living standard [16]. Lastly, the societal level looks at the broader cultural principles and values impacting the other three levels of the framework [14].

### 2.1 Individual risk factors

Chief among those factors are couples' lower educational qualifications [[17], [18], [19], [20], [21], [22]], women's unemployment [22,23], younger age of couples [22,24], and women's early age of marriage [16]. However, a multi-country study found that women's age had no conclusive patterns in IPV [25]. Other studies observed that a significant individual risk factor associated with the perpetration of IPV is witnessing or experiencing abuse in childhood in one's family [[26], [27], [28]].

### 2.2 Family relationship risk factors

The extant literature found that stress factors (e.g., a large number of living children) increase the likelihood of abusing women in some Asian and African countries [20,26,29,30]. Likewise, large family sizes were found to be a risk factor that increased the potential for women experiencing IPV in Jordanian and Palestinian settings [31]. Besides, husbands’ controlling behavior was reported to be a risk factor that influenced marital conflicts and wife abuse in some Arab and non-Arab settings [7,17]. Precisely, imbalances in power dynamics between couples in decision-making were found to increase the possibility of spousal abuse in North American systems [16], EU states [18], and some traditional societies influenced by cultural practices, such as Bangladesh and Jordan [19]. For instance, the number of married women experiencing IPV increased when their participation in household decision-making was low in some traditionally conservative societies [20,31]. More so, a study undertaken in different transitional countries with political and economic transformations in Eastern Europe, Central Asia, and the Caucasus reported contradictory findings [32]. In the study, women with greater economic power and muscles than their partners still experienced IPV [32]. Also, a survey of 28 low- and middle-income countries resulted in mixed findings concerning asset ownership in IPV occurrence, as this may be challenging and protective for IPV against women [25].

### 2.3 Community risk factors

As documented, some studies found associations between poverty, disadvantaged communities, and domestic violence. For instance, a study reported that individuals dwelling in underprivileged communities are prone to experiencing violence in their relationships [17,[19], [20], [21], [22]]. Equally, women living in poor households are most susceptible to spousal violence in some Arab settings [10]. Nonetheless, research in sub-Saharan countries showed that disadvantaged communities and poor households could be protective or risk causes of violence against wives despite the use of similar indicators of economic status or household income levels [33]. Also, studies in three Asian societies found a significant correlation between spousal violence and this factor [34]. Nevertheless, the direction and magnitude of the relationship was different across those societies. In the same vein, a study of five countries in transition reported that the impact of this factor on the domestic abuse of wives was significant only in one country [32]. Other research indicated mixed findings regarding the influence of place of residence on violence against married women. For example, women who live in large cities and urban areas were found to be more likely to be victimized than their counterparts living in small towns or countryside environments [16,22,27]. Other research indicated that women who reside in the countryside experienced a higher level of domestic abuse than those living in urban community contexts [35].

### 2.4 Societal risk factors

From the literature reviewed, a critical risk factor at the societal level is the acceptance of male dominance influencing women's willingness to tolerate violence and consider the experience private [6]. However, some research found mixed findings on the impact of violence acceptance and its justification for spousal abuse in different contexts. For instance, husbands and wives who believe in justifying violence for one or more reasons are highly prone to being abused or being an abuser [21,36]. Further, a review study indicates that men's justification for one or more violent situations covaries strongly with the greater probability of their wives being beaten in different settings [37]. In the same vein, an empirical study on five countries in transition in Eastern Europe, Central Asia, and the Caucasus indicated that women living in communities with acceptance of violence are more likely to sustain domestic violence [32]. Conversely, in more traditional countries, spousal violence was found to be lower in societies characterized by conservative traditions and beliefs about gender or by higher financial dependence on husbands [32]. Also, a Chinese regional study showed that the influence of patriarchal ideology is equivocal [35]. Within Arab contexts, acceptance of wife beating increases the chance of being a victim of each type of IPV against women, except sexual violence [7]. Besides, results from a recent study found that women who justify violence against themselves are more prone to suffer from violence [38]. Apart from spousal violence acceptance justification, it is reported that adult and younger men with more strong feelings about masculinity are more prone to abuse their partners [39].

The empirical literature and research reviewed above suggest the need for embracing an integrative framework with elements to understand different causative conditions of violent attitudes toward women. Hence, this study adopts the socio-ecological conceptualization to gain insights into the victimization of married women in Jordanian society.

## 3 Methodology

### 3.1 Data source and sample

This study uses data generated as parts of the JDHS 2012, which is a national family survey in Jordan. The survey covers all Jordanian governorates, urban and rural localities, and the most disadvantaged regions in Jordan (i.e., the Badia and the Palestinian refugee camps). Administration of the questionnaire survey had two phases: The first phase was a stratification random sampling for selecting the sample where 806 clusters were selected. The second phase involved random sampling administered for selecting a specific number of households (20 in all) in each cluster (DOS & ICF International, 2012). Consequently, 16,120 residential households were selected, resulting in 11,000 wives aged 15–49 years were interviewed (DOS & ICF International, 2012). The domestic violence portion of the survey covered only a sub-sample of only 66 percent the JPFHS cluster selected for the survey. This resulted in sample size to 7027 (DOS & ICF International, 2012). Finally, a method of listwise deletion techniques was used to remove missing data. This has resulted in generating a sample size of 6312 for the purpose of this study. We have not sought any ethical approval because our study uses secondary data generated by the 2012 JDHS. The Department of Statistics in Jordan has obtained ethical approval for JDHS surveys and has generously granted access to the data collected.

### 3.2 How are variables of the study measured?

#### 3.2.1 Dependent variables

The current study examines the IPV experience of women between ages 15 and 49 in the past 12 months when the survey was administered. The married women as respondents were asked to respond to the questions listed below to determine the degree of their experience with ferocious psychological, physical, and sexual attitudes from their husbands. For physical assaults, they were asked to indicate if their husbands have ever:

a. pushed, shaken, or thrown something at them.
b. slapped them.
c. punched or hit them with something harmful.
d. kicked or dragged them.
e. strangled or burnt them.
f. threatened them with a knife, gun, or other weapon.
g. arm-twisted them over the previous year.

For psychological violence and issues, they were asked to indicate whether their husbands have ever.

a. humiliated them.
b. threatened them.
c. insulted them.

The respondents were also asked to indicate if they had ever been forced by their husbands to engage in unsolicited sex. The responses were coded 1 for Yes and 0 otherwise. Cronbach alpha reliability value is 0.84 for physical violence items and 0.72 for the emotional violence questionnaire items.

#### 3.2.2 Independent variables

Those variables are obviously derived from social-ecological framework. Table 1 displays those variables together with their measurements. Those variables are either categorical or binary.

**Table 1:** Independent Variables and their Measurements.

Individual Level			
Name of Independent Variable	Variable Type Categorical Binary		Sub-Categories
Wife Education Husband Education	X X		No education Primary Secondary Higher
Wife's Age Husband Age	X X		5-year age cohorts from 15 to 19 through 45-49 5-year age cohorts from less 25 through above 50
Wife Working		X	1 if yes, 0 otherwise
Wife Experiencing Father Beat Mother		X	1 if yes, 0 otherwise
**Family Level**			
Wife Afraid of Husband	X		Not Afraid Afraid Most of Time Sometimes Afarid
Wife Age at Marriage	X		Less than 18 Years 18–23 Years 24–30 Years Over 30 Years
Husband Controlling Behavior	X		Husband - Being jealous - Insisting where wife going -Accusing wife of infidelity -Not allowing wife to meet female friends -Restricting wife's contact with family members
Number of living children	X		-No child, −1 child −2 children −3 children −4 or more children
House Ownership	X		-Wife sole owner -Wife owns with husband -Husband sole owner
Land Ownership	X		-Wife sole owner - Wife owns with husband -Husband sole owner
Purchase Decisions	X		-Made by wife alone -Made jointly -Made by husband alone
Healthcare Decisions	X		-Made by wife alone -Made jointly -Made by husband alone
Visit Decisions	X		-Made by wife alone -Made jointly -Made by husband alone
**Community Level**			
Region	X		-Central -Northern -Southern
Residence	X		-Urban -Rural
Wealth	X		-Poorest -Poorer -Middle -Rich -Richest
Living in Badia		X	1 if yes, 0 otherwise
**Societal Level**			
Societal Acceptance of Wife Beating		X	1 if yes, 0 otherwise

## 4 Data analysis

Summary statistics were calculated for the frequency of three forms of violence against wives. The study also used three multivariate logistic regression models, based on SPSS, to ascertain whether the risk factors identified above as components of the social-ecological model would predict the three forms violence against married women in Jordan.

### 4.1 Descriptive statistics

Table 2 displays the incidence of the three components of domestic violence sustained by the respondents. Unbracketed numbers are the frequencies; bracketed ones are the relative frequencies or percentages. As Table 2 shows, most of the respondents have not experienced any of the three types of violence. Thus, roughly 17 %, 11 %, and 6 % sustained emotional, physical, and sexual violence, respectively.

**Table 2:** Frequency and relative frequency physical, emotional and sexual violence.

Violence	Category	Frequency	Total
	Yes	No	
Physical	664 (10.5)	5648 (89.5)	6312 (100)
Emotional	1040 (16.5)	5272 (83.5)	6312 (100)
Sexual	359 (5.7)	5953 (94.3)	6312 (100)

### 4.2 Logistic regression results

This data analysis part showcases findings from the three multivariate logistic regression models. As earlier noted, the risk-prone factors of the social-ecological model are used as predictor variables in these three models. The results of these models are presented in Table 3. Each predictor comes with an estimated odd ratio (OR) and whether it is statistically significant at 0.05, 0.01, or 0.001. These statistical significance levels are indicated by asterisks explained under the table. In addition, each predictor comes with a 95 percent confidence interval (95 % CI). The OR associated with each predictor variable is an adjusted OR. This means that the OR of any predictor variable is estimated while controlling for other predictor variables. An OR with a value less than 1.00 suggests the factor in question reduces the likelihood of the perpetration of IPV. In other words, it is prone to protect wives. Conversely, an OR value of more than 1.00 suggests that the factor positively affects or enhances the probability of the perpetration of IPV. In both cases, whether the effect is random or systematic depends on the associated statistical significance level. If the adverse impact is systematic, the associated predictor protects women against violence. On the other hand, if the positive effect is systematic, this indicates that the predictor in question is a risk factor that leads to IPV.

**Table 3:** Logistic regression results.

CI	Physical Violence		Emotional Violence		Sexual Violence	
	OR	95 % CI	OR	95 % CI	OR	95 %
Individual Factors						
Wife Educational Attainment						
Primary	1.025	(0.503, 2.086)	1.066	(0.6, 1.893)	1.216	(0.512, 2.889)
Secondary	1.159	(0.598, 2.244)	1.03	(0.606, 1.751)	1.265	(0.562, 2.849)
Higher	1.038	(0.511, 2.107)	1.096	(0.622, 1.933)	1.593	(0.668, 3.8)
Husband Educational Attainment						
Primary	1.649	(0.676, 4.026)	1.483	(0.728, 3.023)	2.545	(0.817, 7.922)
Secondary	1.216	(0.506, 2.921)	1.335	(0.665, 2.677)	1.678	(0.545, 5.162)
Higher	0.97	(0.387,2.428)	1.349	(0.653, 2.786)	1.05	(0.322, 3.421)
Wife Employed	1.095	(0.796, 1.507)	1.106	(0.861, 1.421)	0.823	(0.545, 1.243)
**Wife's Father Beats Her Mother**	2.268^c^	(1.827,2.815)	2.203^c^	(1.832, 2.649)	1.719^c^	(1.302, 2.269)
**Wife Age**						
15-19	1.298	(0.612, 2.754)	1.259	(0.616, 2.572)	1.179	(0.409, 3.402)
20-24	1.274	(0.573, 2.836)	1.467	(.696, 3.091)	1.566	(0.514, 4.771)
25-29	1.491	(0.634, 2.686)	1.475	(.671, 3.242)	1.245	(0.381, 4.069)
30-34	0.906	(0.343, 2.397)	1.221	(0.51, 2.924)	0.891	(0.242, 3.28)
35-39	0.777	(0.274,2.203)	0.846	(0.337, 2.127)	0.855	(0.219, 3.342)
Husband Age						
25 Years or less	0.644	(0.386, 1.072)	0.827	(0.516, 1.324)	0.572	(0.288, 1.137)
26-30	0.701	(0.411, 1.197)	0.777	(0.475, 1.27)	0.876	(434, 1.768)
31-35	0.537^a^	(0.3, 0.963)	0.764	(0.451, 1.294)	0.943	(0.442, 2.012)
36-40	0.509^a^	(0.264,984)	0.749	(0.417, 1.346)	1.106	(0.476, 2.569)
41-45	0.621	(0.301, 1.28)	0.972	(0.517, 1.824)	1.595	(0.642, 3.962)
46-50	0.331^b^	(0.149, 0.734)	0.836	(0.427, 1.633)	1.1	(0.418, 2.896)

Family Factors						
Number of Children						
Single Child	1.7^a^	(1.034,2.7970	2.08^c^	(1.355, 3.195)	1.042	(0.58, 1.873)
2Children	1.804^a^	(1.102, 2.954)	2.1^c^	(1.374, 3.21)	0.894	(0.501, 1.593)
3Children	1.854^a^	(1.107, 3.104)	2.054**	(1.317, 3.204	0.813	(0.443, 1.492)
4-More Children	2.009^a^	(1.175,3.435)	2.343^c^	(1.484, 3.699)	0.965	(0.522, 1.785)
Wife Afraid of Husband						
Afraid Most of Time	5.197^c^	(3.799, 7.11)	4.306^c^	(3.27, 5.67)	2.8^c^	(1.907, 4.112)
Sometimes Afraid	2.531^c^	(2.016, 3.176)	2.121^c^	(1.767, 2.546)	1.248	(0.928, 1.677)
Wife Marriage Age						
Less than 18 years	0.909	(0.694, 1.191)	1.028	(0.814, 1.296)	1.281	(0.997, 1.829)
18-23	0.965	(0.5651, 1.43)	1.111	(0.803, 1.537)	1.183	(0.717, 1.954)
24-30	1.395	(0.623, 3.125)	1.242	(0.65, 2.375)	1.241	(0.484, 3.186)
Decisions on Purchases						
Made by wife alone	0.869	(0.649, 1.164)	0.723^b^	(569, 0.917)	0.862	(0.601, 1.235)
Made Jointly	1.089	(0.796, 1.489)	0.813	(0.624, 1.059)	0.88	(0.591, 1.309)
Decisions on Visits						
Made by wife alone	0.686^b^	(0.533, 0.883)	0.723	(0.569, 0.917)	0.578^c^	(0.42, 0.797)
Made jointly	0.675*	(0.491,0.928)	0.813	(0.624, 1.059)	0.732	(0.49, 1.093)
Decisions on Healthcare						
Made by wife alone	0.643^c^	(0.507, 0.816)	0.741^b^	(0.61, 0.901)	0.947	(0.696, 1.289)
Made jointly	1.033	(0.756, 1.411)	0.806	(0.609, 1.067)	1.292	(0.726, 1.643)
Husband Controlling Behavior						
Becomes jealous	0.79	(0.609, 1.025)	1.157	(0.931, 1.439)	0.84	(0.609, 1.16)
Accuses partner of infidelity	3.01^c^	(1.934, 4.684)	2.682^c^	(1.75, 4.109)	3.729^c^	(2311, 6.016)
Not permitting partner to meet female friends	2.161^c^	(1.659, 2.814)	2.142^c^	(1.704, 2.692)	1.264	(0.88, 1.817)
Limiting partner's contact with family	2.151^c^	(1.586, 2.916)	2.361^c^	(1.803, 3.093)	1.842^b^	(1.246, 2.723)
Insisting on knowing where wife going	1.295*	(1.038, 1.615)	1.544^c^	(1.289, 1.849)	1.524^b^	(1.145, 2.028)
Wife Land Ownership						
Owns alone	1.009	(0.545, 1.868)	1.542	(0.986, 2.413)	0.708 (0.311, 1.611)	
Owns jointly	1.376	(0.817, 2.317)	1.042	(0.663, 1.636)	1.744	(0.984, 3.093)
**Wife House Ownership**						
Owns alone	1.283	(0.657, 2.503)	0.87	(0.485, 1.558)	2.29^a^	(1.142, 4.589)
Owns jointly	0.872	(0.437,1.739)	0.873	(0.497, 1.534)	0.855	(0.352, 2.077)

Community Factors							
Living in Refugee	Camps	1.748^c^	(1.6, 2.424)	1.288	(0.956, 1.733)	1.183	(0.761, 1.84)
**Living in Badia**	1.039	(0.728, 1.484)	1.094	(0.819, 1.46)	0.556^a^	(0.339, 0.91)	
Region							
Central	1.034	(0.81, 1.321)	0.974	(0.794, 1.195)	1.01	(0.746, 1.366)	
Northern	1.032	(0.79, 1.349)	1.019	(0.819, 1.268)	1.08	(0.807, 1.455)	
Residence							
Urban	1.49^b^	(1.24, 1.75)	0.84	(0.68, 1.037)	1.198	(0.876, 1.64)	
Wealth/Poverty Index							
Richer	1.083	(0.683, 1.717)	0.904	(0.624, 1.31)	0.581	(0.316, 1.07)	
Middle	0.807	(0.559, 1.166)	0.869	(0.647, 1.168)	0.51^b^	(0.319, 0.817)	
Poorer	1.04	(0.768, 1.409)	1.001	(0.776, 1.292)	0.799	(0.552, 1.156)	
Poorest	1.218	(0.923, 1.607 1.112)	(0.876, 1411)	0.74 (0.522,1.049)			

Social Factors							
**Acceptance of Wife Beating**	1.337^a^	(1.068, 1.674)	1.105	(0.91, 1.341)	1.37^a^(1.031, 1.82)		

a p <.05.

b p < .01.

c p < .001.

#### 4.2.1 Individual factors

From the data analyzed, most individual factors did not significantly influence respondents experience with domestic violence. However, age in marriage determines the extent to which women are likely to experience IPV. As shown in Table 3, husbands whose ages fall within the age cohorts of 31–35 years, 36–40, and 46–50 years are unlikely (P˂ 0.05 and P˂ 01) to perpetrate physical violence against their wives. In other words, these ages are protective factors against wife physical victimization. An unexpected finding is that wives' educational attainment and working status are not statistically significant predictors of IPV. Similarly, husband's educational attainment level is not a statistically significant explanatory variable for domestic abuse against wives.

On the female victim's side, a statistically significant risk factor is witnessing her father beating her mother (P˂0.001). This is a crucial individual risk factor; such beating goes up by 2.268 times the possibility of husbands abusing their wives. Furthermore, this factor predicts the likelihood of ferocious men's attitudes emotionally and sexually toward their wives at P˂ 0.001 and P <0.001, respectively.

#### 4.2.2 Family risk factors

In the context of intra-family relationships, some factors exhibit statistically significant covariation with all forms of IPV. Of particular importance is wife being scared of her partner most of the time and sometimes afraid (P˂0.001 and P˂0.001, respectively). Wife being scared most of the time increases by five times the chance of being physically abused (P˂0.001). Similarly, this factor has a statistically significant relationship with both emotional and sexual aggression against wives (P˂0.001). Another key family risk factor is the number of children in the family unit. All four categories associated with this factor are statistically significant causative factors of physical and emotional abuse experienced by women (*P*-values <0.05, <0.01, <0.001).

A third critical factor leading to aggression against wives is husbands’ propensity to be controlling. All four behavioral components of this factor are statistically significant (P˂0.001) correlates of IPV sustained by wives. Those married women whose husbands accuse them of infidelity are at 3.01 and 3.7 times the risk of, respectively, physical violence and sexual violence. These components are also statistically significant predictors (P˂0.001) of psychological and sexual abuse inflicted on wives.

Decisions on purchases made by wives alone appear to protect them (P˂0.01) against their husbands inflicting emotional aggression on them. Similarly, decisions on visits made by wives alone are likely to protect them against physical abuse (P˂0.01) and sexual victimization (P˂0.001). Healthcare decisions made by wives alone appear to protect them from physical aggression (P˂0.001) and psychological victimization (P˂0.01). Interestingly, collaborative decisions are not statistically significant predictors of IPV, except visit decisions (P˂0.05). House ownership exposes wives to sexual aggression if they own a house alone (P˂0.05).

#### 4.2.3 Community risk factors

From the data, a wife living in a refugee camp can potentially suffer from only physical aggression (P <0.001). In contrast, there is a reduced likelihood of sexual aggression toward wives in the Badia community (P <0.05). This factor appears to be a protective one. Similarly, a wife living in an urban area can suffer from physical aggression from her partner (P˂0.01). Household levels of wealth/poverty tend to expose wives to sexual attacks. However, none of those levels is a statistically significant causative factor of physical violence against domestic female partners. The only exception is the middle level (P <0.01), which appears to protect women against this kind of aggression.

#### 4.2.4 Societal risk factors

Acceptance of wife-beating is statistically significant as a causative factor due to male dominance in Jordanian society. This patriarchy-influenced practice can lead to physical aggression (P˂0.05) and sexual aggression (P˂0.05) committed against wives by their partners.

## 5 Discussion

As previously noted, most predictor variables representing the six risk-prone factors at the micro or individual level are not statistically significant predictors of IPV. For instance, a wife's educational attainment, working status, and wife's age at marriage are not statistically substantial covariates of IPV. This finding contradicts recent literature that reports spouses' education and employment as protective or risk factors for IPV [17,18,23]. A recent large-scale study covering 28 countries reports that women's age does not appear to have a conclusive pattern in IPV [25]. Similarly, an earlier study reports that women's age is a risk factor influencing their experience with IPV only at a minimal or weak level [7]. However, the findings that the youngest and oldest age cohorts of wives are not statistically significant predictors of spousal violence are consistent with recently reported research results. It is also worth noting that husbands' physical, emotional, and sexual victimization of wives, who witnessed their fathers abuse their mothers, is consistent across several studies reporting findings from different settings, including Arab societies [26,27]. In this study, this finding is no exception. Jordanian wives who reported witnessing their mothers being abused by their husbands were themselves physically, emotionally, and sexually victimized by their husbands. Thus, families in Jordanian society need to rethink the process through which the younger generations are socialized. For instance, they must educate them to embrace dialogue instead of venting anger violently when resolving differences. Besides, parents need to ensure discrepancies are settled amicably for the younger generations to imbibe such a peaceful philosophy, including treating other people as one expects their family to be treated.

This study's findings on family risk factors are particularly interesting. According to a study, the family level in the social-ecological conceptual framework focuses on family-based relationships, particularly structural roles and changing forms of engagement [7]. Role structure and interactional dynamics are reflected in all family-level-related variables, including women's economic empowerment indicated by owning assets, their participation in family decision making, husbands' controlling behavior, wives' being afraid of husbands, and the presence of children. Indeed, several family dynamics as risk factors for the perpetration of IPV are reported in a recent study in Bangladesh [40]. A key finding that emerges from this study is that a wife's decision-making power, operationally defined as a wife alone making decisions on purchasing family needs or visits to family and relatives, reduces the probability of her sustaining physical and psychological violence inflicted by her husband. The findings, therefore, indicate that, indeed, there is a power imbalance in couples' relationships and decision-making abilities [18,19]. As such, stakeholders, including families, state officials, and individuals, may consider promoting such principles at different societal levels to foster a culture of respect for human dignity. Families in Jordanian society should encourage women's economic emancipation and active involvement in decision-making. However, women must also be educated to appreciate their spouses, engage with them respectfully, and avoid attitudes that may lead to a lack of trust in their marital relationships.

This study reports that wives' possession of assets is a risk-prone factor that exposes them to psychological violence when they own lands alone. Further, they are prone to sustain sexual violence committed by their husbands when they jointly own land. Husbands' abuse of economically empowered wives is probably explicable in their attempts to maintain their dominant status in the established family power structure. Empowered wives are perceived to challenge the power imbalance that favors husbands. Given the patriarchal nature of Jordanian society, husbands resort to some means to maintain their dominance, perceived to be threatened by wives' empowerment, in the family power structure should come as no surprise. As noted elsewhere, the abuse inflicted on empowered wives is a resource utilized by abusive husbands to maintain the power imbalance that favors them [7]. In this study, all husband-controlling behavior variables are statistically significant covariates of all forms of IPV except for husband jealousy, a statistically significant predictor of psychological IPV. This result accords with previous research results in various settings. As a characteristic of patriarchal societies [41], husband's controlling behavior results from a male predisposition to exercise dominance over females. In family relations, it is practiced to solve marital conflicts. In the Jordanian patriarchal society, husband's proclivity to control their partners is probably rooted in the husband's beliefs in rigid traditional gender roles characteristic of such a society. As previously noted, the fear of wives of their husbands increases the probability of those wives experiencing all forms of IPV. However, this fear may not fully account for the IPV perpetrated against wives. It may be due to several factors, including marital conflicts, husband-controlling behavior, alcohol abuse, unemployment, emotional problems (e.g., anger and hostility), lower status, and traditional sex-role beliefs. It can also result from conflicts over children, sex, money, and social activities that may enhance imbalance in the family power structure.

As noted in the literature, the community component in the social-ecological model operates at a broad community level that includes localities with socio-economic features, particularly poverty and its attendant standard of living [14]. In this study, locality exposes married women to violence inflicted on them by their partners [32]. Thus, wives residing in Jordanian urban localities are more prone to suffer from physical abuse perpetrated by their partners. In contrast, living in Badia protects wives from sexual violence by their husbands. As expected, wives living in disadvantaged areas (i.e., refugee camps in this study) are at an increased risk of being physically victimized by their partners. This result accords with some empirical studies covering the Arab context [[18], [19], [20], [21]]. Thus, community leaders and the government need to provide some safety nets to alleviate hardships and empower those living in refugee camps.

A noteworthy finding of this study is that society's acceptance of wife-beating by male partners is a systematic cause of the physical and sexual abuse of female partners [21,35]. This societal dimension underscores the place of culture and tradition when using the framework to explore IPV [14]. In the Jordanian cultural context, this acceptance and its attendant victimization of wives indicate the existence and influence of traditional values alongside modern secular values in present-day Jordan. As part of the broader Middle Eastern Arab societies undergoing transition, the Jordanian context reflects the lingering impact of patriarchy on family relations, even as the community is transitioning to modernity. Put differently, contemporary Jordanian society is still characteristically patriarchal insofar as family relations are concerned.

## 6 Conclusions, implications, limitations, and future research

In the Jordanian context, several factors at the individual, family, community, and societal (i.e., cultural values and beliefs) levels interact to engender all forms of domestic abuse against Jordanian female partners. According to our study, societal and family factors are the most critical causes of violence sustained by those partners. Jordanian women's economic and social efficacy seems to protect them against violence perpetrated by their husbands. Furthermore, the patriarchal factor is still valid for the emotional, physical, and sexual victimization of female partners in the Jordanian cultural context. Thus, the study has some implications for stakeholders in Jordanian society, including family, community, and government. For instance, as the most important agent of socialization, the family must engage in a reorientation drive to ensure that male and female persons are empowered, treasured, and appreciated regardless of their educational qualifications. In collaboration with the government, they must also provide access to social-cultural contexts [42] where young people are offered Islamic education to stimulate righteous attitudes grounded in Islamic principles [43]. Through such exposure, younger generations can also be provided personalized support to develop entrepreneurial skills for escaping poverty. Given the essential roles of women in society, family, community, and government must continue to explore strategies to support people in rural communities and non-economically capable neighborhoods. These will include helping them economically by funding entrepreneurial and small-scale businesses, providing equitable access to educational programs, and launching more awareness campaigns regarding the negative impact of IPV on communities. Religious values emphasizing the need to embrace tolerance, respect diversity, and appreciate everyone to foster peaceful co-existence should also be taught at home and educational institutions [41]. Lastly, the government should ensure that policies for discouraging IPV are made and enforced and those culpable are brought to justice. Doing this will send a solid signal to individuals, families, and communities with the potential to support IPV that they need to rethink and promote peaceful engagement among one another.

Our study has three limitations. First, it is a cross-sectional study, which, by definition, does not capture the variation in the values of a variable that occur over span of time. Another limitation is that some independent variables mentioned in the literature on IPV, seen as relevant from a social-ecological perspective, are not found in the data used in this study. Examples of such include partners' relationships, abusive female partners, and detailed community features. A third limitation has to do with the treatment of individual perceptions as societal and community factors. This limitation applies to such factors as society's acceptance of wife beating and the extent of poverty/wealth involved. Individual or respondent's perception of societal acceptance of wife beating is not, obviously, a true direct measure of such acceptance. However, it may be taken as an indirect measure insofar as a it is reflection or the result of an internalized cultural norm on the part of individuals. By the same token, individual perceptions of wealth/poverty levels may also be taken as a reflection of some internalized reference categories of wealth/poverty prevalent in the community. Nonetheless, the study contributes to the existing corpus of knowledge in identifying different risk-prone factors, at all levels of the social-ecological framework, associated with violence sustained by female partners.

Future research needs to use a longitudinal framework to capture changes over time. In addition, it should incorporate relevant independent variables such as religion and, drinking alcohol, and other psychological factors related to the personality of the spousal partner, not hitherto included in current research, that crops up in the literature on IPV. Further, incorporating qualitative data analysis into IPV research will strengthen our understanding of the results of quantitative analysis. Finally, future IPV researchers should specify the implications of their research for general human development programs, woman empowerment policies, and what can be done to raise awareness of IPV, mainly where the prevalence of the domestic abuse of female partners is alarming or particularly marked.

## Data availability

The authors do not have the authorization to share the data. Interested researchers may contact the Department of Statistics, Macro International, and ICF International in Jordan to access the data.

## CRediT authorship contribution statement

**Hussein Mohammad Alothman:** Writing – review & editing, Writing – original draft, Project administration, Methodology, Formal analysis, Conceptualization. **Abdel Rahman Ahmed AbdelRahman:** Writing – review & editing, Writing – original draft, Methodology, Formal analysis, Conceptualization. **Semiyu Adejare Aderibigbe:** Writing – review & editing, Writing – original draft, Conceptualization. **Muhamed Ali:** Writing – review & editing, Writing – original draft.

## Declaration of competing interest

The authors declare that they have no known competing financial interests or personal relationships that could have appeared to influence the work reported in this paper.
